# Predictors of Knowledge and Risk Perception on Hepatitis B Virus Infection Among People Who Inject Drugs in Dodoma, Tanzania: A Cross-sectional Study

**DOI:** 10.24248/eahrj.v9i2.844

**Published:** 2025-12-24

**Authors:** Rabi Msabi Sumbuka, Erick Donard Oguma, Stephen M. Kibusi

**Affiliations:** a Department of Clinical Nursing, St. Gasper College of Health and Allied Science, Itigi-Singida, Tanzania; b Department of Clinical Nursing, School of Nursing and Public Health, University of Dodoma, Dodoma, Tanzania; c Department of Public Health and Community Nursing, School of Nursing and Public Health, University of Dodoma, Dodoma, Tanzania

## Abstract

**Background:**

Globally, an estimated 15.6 million individuals inject drugs, with about 1.4 million living with HBV infection. People who inject drugs (PWIDs) represent one of the populations most vulnerable to hepatitis B virus (HBV) infection due to frequent exposure to contaminated injecting equipment and limited access to preventive and treatment services. Despite this high vulnerability, a substantial gap persists in knowledge and risk perception regarding HBV infection transmission and prevention among PWIDs. This study aimed to assess the predictors of knowledge and risk perception toward HBV infection among PWIDs.

**Methods:**

A facility-based analytical cross-sectional study was conducted from June to September 2021 at the Itega Methadone Clinic in Dodoma, Tanzania. A total of 206 participants were selected through simple random sampling. Data were collected using a structured questionnaire assessing knowledge and risk perception toward hepatitis B virus infection. Binary logistic regression analysis was performed to identify predictors of poor knowledge and low risk perception, with statistical significance set at *p* <.05 and 95% confidence intervals (CI).

**Results:**

Overall, knowledge about hepatitis B virus (HBV) transmission and prevention among people who inject drugs (PWIDs) was poor, with only 36% demonstrating adequate knowledge. Inadequate knowledge was significantly associated with low education level (AOR 2.44; 95% CI, 1.29–4.68; *p*=.005), short duration of methadone treatment (<1 year) (AOR 2.26; 95% CI, 1.12–4.55; *p*=.023), and low economic status (≤ TZS 100,000) (AOR 3.93; 95% CI, 1.01–15.25; *p*=.048). Regarding risk perception, the majority of participants underestimated their susceptibility to HBV infection, with only 25% perceiving themselves at high risk. Low risk perception was strongly predicted by low education level (AOR 12.68; 95% CI, 3.44–46.83; *p*<.001) and inadequate knowledge (AOR 2.63; 95% CI, 1.28–5.39; *p*=.008).

**Conclusion:**

This study revealed that knowledge and risk perception on hepatitis B virus infection among PWIDs remain alarmingly low. Only one-third of participants demonstrated adequate knowledge, and merely a quarter perceived themselves at high risk of infection. Low education level, economic disadvantage, and shorter duration of methadone treatment were the strongest predictors of poor HBV knowledge, while low risk perception was significantly associated with both low education and poor knowledge. These findings underscore the urgent need to integrate comprehensive HBV education, counselling, and vaccination awareness into harm-reduction and methadone programs. Interventions should particularly target newly enrolled clients and those with limited formal education or low income to foster accurate risk perception and improve preventive behaviours.

## BACKGROUND

Globally, an estimated 254 million people are living with Hepatitis B virus (HBV) infection in 2024, with about 2.2 million new infections occurring each year, the majority through mother-to-child transmission or early childhood exposure. HBV infection is responsible for nearly 1.1 million deaths annually, primarily due to cirrhosis and liver cancer. The African Region accounts for approximately two-thirds of all new infections, reflecting persistent gaps in vaccination coverage, testing, and access to treatment in low- and middle-income countries.^1^ In Tanzania, findings from the Tanzania HIV Impact Survey (THIS 2022-2023) showed that the prevalence of HBV infection among adults aged 15 years and above was 3.5%, with regional variations ranging from 1.8% in Mtwara to 6.9% in Rukwa. The prevalence was higher among individuals living with HIV (6.2%) compared with those who were HIV negative (3.4%).^2^

People who inject drugs (PWIDs) are among the populations at highest risk for HBV infection due to frequent sharing of contaminated needles, syringes, and other injection equipment.^3–7^ Injecting practices facilitate direct blood-to-blood transmission of HBV, while limited access to preventive services, vaccination, and testing further increases vulnerability.^8,9^ Studies have consistently shown HBV prevalence among PWIDs to be several times higher than in the general population,^5,7,10^ underscoring the need for integrating HBV prevention, screening, and treatment within harm-reduction and methadone programs.

An estimated 15.6 million people inject drugs globally, among whom 1.4 million were infected with Hepatitis B viral infection.^3^ In Tanzania, the reported prevalence of HBV infection among PWIDs in the general population is approximately 7.8%, indicating a substantially higher burden compared with the national adult prevalence of 3.5% reported in the THIS 2022-2023.^2,11^

The World Health Organization (WHO) has set ambitious global targets to eliminate viral hepatitis as a public health threat by 2030. These targets aim to achieve a 90% reduction in new HBV infections and a 65% reduction in HBV-related mortality compared with 2015 levels. To reach these goals, WHO calls for ensuring that 90% of infants receive the hepatitis B birth dose, 95% complete the full vaccination series, 80% of people with chronic HBV infection are diagnosed, and 80% of those eligible receive antiviral treatment. The Global Hepatitis Report 2024 highlights that while substantial progress has been made in vaccine coverage, testing and treatment uptake remain far below targets, particularly in low- and middle-income countries, including those in sub-Saharan Africa.^1^

The United Republic of Tanzania, through its Ministry of Health, developed the National Strategic Plan for the Control of Viral Hepatitis (2018/19–2022/2023) in response to the growing burden of viral hepatitis infections.^12^ More than 10 billion Tanzanian shillings (TZS) were allocated to support the effective implementation of this plan. The strategy aims to promote advocacy and increase public awareness of viral hepatitis to enhance service utilization; strengthen preventive measures to halt transmission within the population; expand access to diagnostic, care, and treatment services in line with the continuum of care and the principles of universal health coverage; and reinforce strategic information systems and surveillance for monitoring, evaluation, and evidence-based decision-making.

While national strategies have reduced HBV prevalence in some high-risk groups like healthcare workers, the impact on PWIDs remains uncertain. Recent studies indicate a rising HBV prevalence in this population,^13^ potentially linked to inadequate knowledge. Most existing research in Tanzania has focused on prevalence in Dar-es-Salaam,^11,13^ leaving a gap in understanding the predictors, knowledge, and risk perception of PWIDs in Dodoma. This study therefore aims to assess the predictors, knowledge and risk perception towards the transmission and prevention of Hepatitis B viral infection among people who inject drugs at Itega Methadone Clinic in Dodoma.

## METHODS

### Study Setting and Study Design

This facility based analytical cross-sectional study was conducted at Itega Methadone Clinic of Mirembe National Mental Health Hospital in Dodoma Region from June to September, 2021. The clinic serves people who inject drugs (PWIDs) referred from various regions across the country and provides integrated harm-reduction services, including counselling, medical care, and infection prevention education.

### Study Population

The study population comprised individuals enrolled in methadone maintenance treatment at the Itega Methadone Clinic in Dodoma, Tanzania. The study targeted both male and female clients who had a history of injecting drug use prior to treatment enrollment and who regularly attended the clinic during the data collection period.

### Eligibility Criteria

#### Inclusion Criteria

Participants were eligible if they were aged 18 years or older, enrolled in methadone maintenance treatment at the Itega Methadone Clinic, had a history of injecting drug use prior to methadone initiation, were able to communicate clearly, and provided written informed consent to participate.

#### Exclusion Criteria

Participants were excluded if they were under the influence of substances at the time of data collection (exhibiting violent or erratic behaviour), had severe medical or psychiatric illness affecting participation, and were temporarily enrolled on methadone for less than one month.

### Sample Size Estimation

The sample size was calculated using Cochran's formula for a single population proportion: n=Z^2^×P(100−P)/E^2^,^14^ where n is the required sample size, Z is the standard normal value corresponding to a 95% confidence level (1.96), P is the estimated proportion of adequate HBV knowledge, and E is the margin of error (5%). Based on a study conducted in Mwanza, Tanzania, by Kilonzo et al. (2021), which reported that 16.6% of people who injected drugs had adequate knowledge of HBV,^11^ “P” was set at 16.6%. Substituting these values yielded a minimum sample size of 213 participants; after adjusting for possible non-response, a total of 206 participants were enrolled in the study.

### Sampling Procedure

A simple random sampling technique was employed to select participants from the registry of clients receiving methadone maintenance treatment at the Itega Methadone Clinic in Dodoma. A list of all eligible clients was obtained from clinic records and assigned numbers, after which random numbers were generated using a computer-based randomization process to identify participants until the required sample size was reached. This approach ensured that each eligible client had an equal chance of being selected, thereby minimizing selection bias.

### Data Collection Method

Data were collected between June and September 2021 using a pretested structured questionnaire administered through face-to-face interviews in Swahili, the local language. The questionnaire covered participants’ socio-demographic characteristics, knowledge of HBV transmission and prevention, and risk perception of the infection. Trained research assistants with experience in working with key populations conducted the interviews in a private setting within the clinic to ensure confidentiality and comfort.

### Data Collection Tool

A structured questionnaire was adapted from previous study conducted in Mwanza and Nigeria.^15–17^ The questionnaire consisted 9 items of participant demographic information, 7 items of knowledge and 7 items of risk perception towards transmission and prevention of Hepatitis B virus infection. Tool was prepared in English language, then was translated into Swahili language to make it user-friendly to the participants. The final version of the questionnaires was organized into of three parts with a total of 23 questions.

### Validity and Reliability

The questionnaire was developed based on existing literature and validated tools from similar studies conducted among people who inject drugs. Content validity was ensured through expert review by public health researchers and clinicians experienced in hepatitis and harm-reduction programs. The tool was pretested on 10% of the target population at a methadone clinic outside the study site to assess clarity, consistency, and cultural appropriateness. Reliability testing yielded a Cronbach's alpha of 0.82, indicating good internal consistency of the instrument.

### Variables Description and Measurement

#### Independent Variable

The participants’ characteristics such as age, sex, education level, occupation, marital status, economic status, duration methadone maintenance, residence area and ethnicity were the independent variables.

#### Dependent Variable

The dependent variables were “Knowledge” and “Risk perception” on HBV infection among injection drug users.

Knowledge was assessed by (7) questions regarding aetiology, risk factors, symptoms, transmission, preventive measures, and vaccination of HBV. The correct response was scored (1) and incorrect response (0). The minimum score was (0) and maximum score was (7), the respondents were considered to have adequate knowledge if they scored (4–7), and were considered to have inadequate knowledge if scored (0–3).

Risk perception on HBV infection was assessed by using seven (7) questions. The correct response scored (1) and incorrect response (0). The minimum score was (0) and maximum score was (7). A cut-off point of ≤3 correctly answered questions was considered to be a low risk perception, whereas >3 correctly answered questions was considered as high risk perception on HBV infection. This cut-off corresponds to achieving less than 50% of the total possible score and has been commonly applied in knowledge and perception scales to distinguish inadequate from adequate levels of risk perception. Using a mid-point threshold allows for a simple, transparent, and interpretable classification.^18,19^

### Data Analysis

Data were cleaned and analyzed using IBM SPSS Statistics for Windows version 26.0 (IBM Corp, Armonk, NY, USA). Descriptive statistics, including frequencies and proportions, were used to summarize the data. Binary logistic regression analysis was performed to identify predictors of knowledge and risk perception on HBV infection among people who inject drugs. The level of statistical significance was set at *P*<.05 with a 95% confidence interval (CI). Variables that showed significant associations in the final regression model were reported using adjusted odds ratios (AORs), corresponding *P* values, and 95% CIs.

### Ethical consideration

Ethical approval for this study was obtained from the University of Dodoma Institutional Research Review Committee (UDOM-IRRC). Permission to conduct the study was also granted by the management of the Itega Methadone Clinic and the Dodoma Regional Medical Office. Written informed consent was obtained from each participant after explaining the purpose, procedures, potential risks, and benefits of the study. Participation was voluntary, and respondents were informed of their right to withdraw at any time without repercussions. To ensure confidentiality, no personal identifiers were recorded, and all data were stored securely and used solely for research purposes.

## RESULTS

### Socio-demographics Characteristics of Study Participants (N=206)

A total of 206 participants were recruited during the data collection with a total responding rate of (96.7%). The mean age of study participants was 33 years, while the minimum and maximum age was 19 years and 62 years, respectively. Most of the people who inject drugs (PWID) were in the young-adult age group (26–35 years).

The majority of participants were male, and more than half were unmarried. Most participants resided in urban areas, while a smaller proportion came from rural settings. The majority identified as Christian, and more than half had attained primary-level education, while only a small minority had never attended school. In addition, more than half of the participants were unemployed, and most reported earning a low monthly income (Tanzanian Shilling (TZS) 50,000 per month).

Regarding treatment characteristics, participants had varying durations of methadone treatment, ranging from a short initiation period (one month) to several years (four years), with an average duration of approximately one year. The majority had been on methadone treatment for less than one year, Table 1.

**TABLE 1:** Describing the Socio-demographics Characteristics of Study participants (N=206)

Demographic Characteristics	Frequency (n)	Percent (%)
Age groups of participants		
18–25 years	46	22
26–35 years	100	49
36–50 years	40	19
Above 50 years	20	10
Sex of participants		
Male	170	83
Female	36	17
Religion of participants		
Christian	136	66
Muslim	70	34
Duration of methadone treatment		
1 month–6 months	60	29
7 months–12 months	83	40
13 months–24 months	43	21
Above 24 months	20	10
Marital status of participants		
Single	136	66
Married	70	34
Residence area		
Urban	175	85
Rural	31	15
Education level of participants		
Never gone to school	9	4.4
Primary level	104	50.4
Secondary level	78	37.9
Higher education	15	7.3
Occupation status of participants		
Student	6	2.9
Employed or self-employed	90	43.7
Unemployed	110	53.4
Reported Monthly Income		
Less TZS 50,000	104	50.5
TZS 50,000–100,000	72	34.9
Above TZS 100,000	30	14.6

### Knowledge of HBV Infection Transmission and Prevention among PWIDs

**FIGURE 1:**
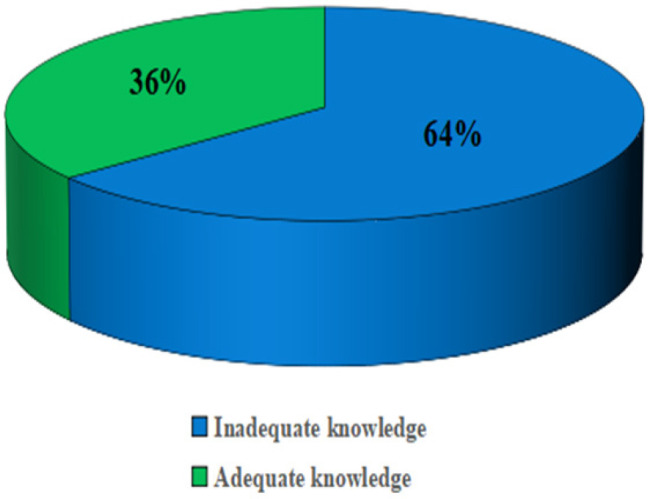
Overall Knowledge of HBV Infection Transmission and Prevention Among People Who Inject Drugs Attending the Methadone Clinic in Dodoma (N=206)

More than half of the participants 132 (64%) had inadequate knowledge and only 74 (36%) had adequate knowledge regarding the mode of transmission and preventive measures of hepatitis B virus infection, Figure 1. The majority of the participants had heard about Hepatitis B virus infection and the methadone clinic. However, more than half did not know the causative agents of Hepatitis B infection, and slightly fewer than half did not know if drug injection was a risk factor for acquiring Hepatitis B infection.

Regarding the mode of transmission, one in three participants reported contact with blood or body fluid of HBV infection carriers, A little more than one in two mentioned sharing piercing equipment such as needle and syringes, about one in fifteen agreed to have had unsafe sexual contact, and one in five reported that they did not know.

Regarding preventive measures and vaccination, only a little more than half of the respondents reported avoiding sharing piercing instruments, one in ten avoiding unsafe sexual contact, close to one in seven reported wearing gloves when handling blood and body fluids, while the majority were not aware of HBV vaccination, Table 2.

**TABLE 2:** Responses to Questions Pertaining to Knowledge of HBV Infection Transmission and Prevention Among PWIDs Attending the Methadone Clinic in Dodoma (N=206)

Statements	Frequency (n)	Percent (%)
Ever heard about hepatitis B		
Yes	180	87.4
No	26	12.6
Where did you hear		
Methadone Clinic	149	72.3
Other places	5	2.5
Mass media	26	12.6
Not applicable	26	12.6
Causative agents of hepatitis B		
Viruses	80	38.8
Bacteria	81	39.4
Don't know	45	21.8
Routes of hepatitis B transmission		
Unsafe sexual contacts	14	6.8
Sharing piercing instruments	92	44.7
Infected blood products and body fluid	60	29.1
Don't know	40	19.4
Does injecting drug a risk factor for acquiring HBV infections?		
Yes	116	56.3
No	90	43.7
Can a hepatitis B patient present without symptoms		
Yes	92	44.7
No	114	55.3
Preventive measures against hepatitis B virus infection		
Avoiding sharing piercing instruments	111	53.9
Avoiding physical contact with infected people	20	9.7
Wearing gloves when handling blood, body fluids	30	14.6
Don't know	45	21.8
Do you know if hepatitis B vaccination can prevent hepatitis B infections?		
Yes	71	34.5
No	135	65.5
What is the recommended vaccination dosage?		
One	10	4.9
Two	5	2.4
Three	56	27.2
Not applicable	135	65.5

### Predictors of Knowledge on HBV Infection Transmission and Prevention among PWIDs

The predictors of knowledge on HBV infection transmission and prevention among PWIDs was assessed using binary logistic regression model. As Table 3 indicates, participants who had low education level (informal and primary education) (AOR 2.44; 95% CI, 1.29–4.68); *P*=.005), were 2 times more likely to have inadequate knowledge on HBV transmission and prevention than those who had high education level. Likewise, the participants who had a short duration of methadone treatment (less or equal to one year) (AOR 2.26; 95% CI, 1.12–4.55; *P*=.023), were 2 times more likely to have inadequate knowledge than those who had been on methadone treatment (greater than one year) much longer. Moreover, the participants who had reported average monthly income of less than TZS 100,000 per month (AOR 3.93; 95% CI, 1.01–15.25; *P*=.048), were 4 times more likely have inadequate knowledge compared to those who reported a monthly income greater or equal to TZS 100,000.

**TABLE 3:** Multivariable Binary Logistic Regression Analysis Showing Predictors of Knowledge on HBV Infection Transmission and Prevention Among PWIDs Attending Methadone Clinic in Dodoma (n=206)

Variables	Knowledge Status		AOR	95% CI		*p-value*
	Inadequate	Adequate		Lower	Upper	
Education status						
Primary and below	67 (59%)	46 (41%)	2.441	1.27	4.68	.005
Secondary and above	65 (70%)	28 (30%)	Ref			
Occupation status						
Employed or Self-employed	24 (27%)	66 (73%)	0.207	0.10	0.42	.064
Unemployed	108 (93%)	8 (7%)	Ref			
Duration of methadone treatment						
≤ One year	93 (64.5%)	51 (35.4%)	2.255	1.12	4.55	.023
>One year	39 (67%)	23 (33%)	Ref			
Reported Monthly Income						
≤ 100,000/= per month	94 (57%)	71 (43%)	3.927	1.01	15.25	.048
>100,000/= per month	38 (93%)	3 (7%)	Ref			
Religion						
Christian	91 (67%)	45 (33%)	0.35	0.17	0.72	.087
Muslim	41 (58.6%)	29 (41.4%)	Ref			

Adequate knowledge^*^ (Reference)

### Risk Perception on Hepatitis B Virus Infection among PWIDs

**FIGURE 2:**
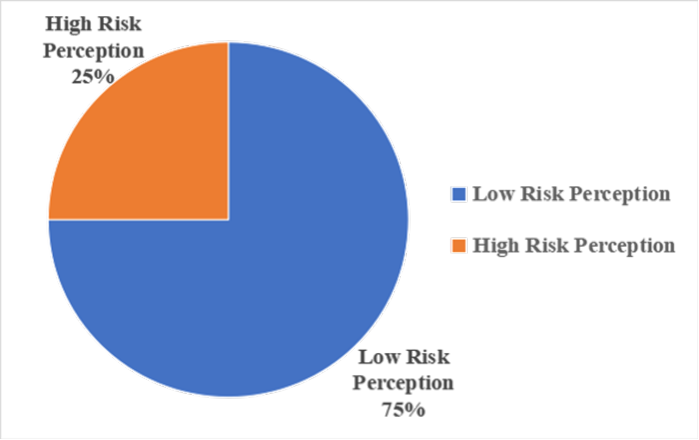
Risk Perception of Hepatitis B Virus Infection Among People Who Inject Drugs Attending the Methadone Clinic in Dodoma, Tanzania (N=206)

Majority of participants had a low risk perception toward hepatitis B virus (HBV) infection, indicating that most did not perceive themselves to be at high risk of acquiring HBV, Figure 2. Similarly, as presented in Table 4, more than half of the participants believed that they were not at risk of being infected with HBV. Nearly half perceived that there was no need for healthy individuals to take preventive measures against HBV infection. Furthermore, about one in five participants perceived that hepatitis B vaccination was not effective, while more than half reported that they did not know whether the vaccine was effective.

**TABLE 4:** Responses to Questions Pertaining to Risk Perception on Hepatitis B Virus Infection Among PWIDs Attending Methadone Clinic in Dodoma (N=206)

Variables	Frequency (n)	Percentage (%)
Do you think HBV infection transmissions are serious to elders and young children only?		
Yes	67	32.5
No	35	17
Don't know	104	50.5
Who do you think can transmit hepatitis B virus to other people?		
Patients	70	34
Any person	78	37.9
Don't know	58	28.1
Do you think you are at risk of hepatitis B infection currently?		
Yes	94	45.6
No	112	54.4
Do you think hepatitis B vaccination is effective on prevention of hepatitis B infections?		
Yes	54	26.2
No	43	20.9
Don't know	109	52.9
Do you think health people need to take preventive measures against hepatitis B infections?		
Yes	108	52.4
No	98	47.6
Do you think hepatitis B vaccination is necessary to you?		
Yes	108	52.4
No	98	47.6
Do you think health education on hepatitis B transmission and prevention is necessary to you?		
Yes	107	51.9
No	99	48.1

### Predictors of Risk Perception on Hepatitis B Virus Infection among PWIDs

The predictors of risk perception on Hepatitis B virus infection among PWIDs were assessed using a Binary logistic regression model. From Table 5, Participants who had a low education level (informal and primary education) (AOR 12.68; 95% CI, 3.44–46.83; *P*<.001) were 12 times more likely to have low risk perception than those who had a high education level (secondary education and above). In addition, the participants who had inadequate knowledge of HBV transmission and prevention (AOR= 2.63; 95% CI, 1.28–5.39; *P*=.008), were 2 times more likely to have low risk perception than those who had adequate knowledge of HBV transmission and prevention.

**TABLE 5:** Multivariable Binary Logistic Regression Analysis Showing Predictors on Risk Perception of Hepatitis B Virus Infection Among PWIDs Attending Methadone Clinic in Dodoma (N=206)

Variables	Risk Perception Status		AOR	(95% CI)		*P Value*
	Low	High		Lower	Upper	
Education status						
Primary and below	88 (78%)	25 (22%)	12.683	3.44	46.83	.000
Secondary and above	67 (70%)	26 (30%)	Ref			
Occupation status						
Employed or Self-employed	72 (80%)	18 (20%)	0.837	0.43	1.64	.603
Unemployed	83 (72%)	33 (28%)	Ref			
Duration of methadone treatment						
≤ One year	100 (81%)	24 (19%)	2.993	2.00	3.84	.052
>One year	55 (67%)	27 (33%)	Ref			
Reported Monthly Income						
≤ 100,000/= per month	131 (84.5%	24 (15.5%)	0.843	0.43	1.65	.618
>100,000/= per month	24 (47%)	27 (53%)	Ref			
Knowledge status						
Inadequate knowledge	96 (73%)	36 (27%)	2.63	1.28	5.39	.008
Adequate knowledge	59 (69%)	15 (31%)	Ref			

High risk perception (Reference)^a^

a Risk perception of hepatitis B virus (HBV) infection was classified into high and low levels, with high risk perception serving as the reference category in the logistic regression analysis.

## DISCUSSION

This study examined knowledge and risk perception toward hepatitis B virus (HBV) infection among people who inject drugs (PWIDs) attending methadone treatment in Dodoma, Tanzania. Overall, findings revealed that both knowledge and risk perception were low, with only one-third of participants demonstrating adequate knowledge of HBV transmission and prevention, and merely one-quarter perceiving themselves at high risk of infection. These results highlight a critical public health gap that may contribute to continued vulnerability and low uptake of preventive services such as screening and vaccination among PWIDs.

Low education level, short duration of methadone treatment, and low economic status were significant predictors of poor knowledge. The association between limited education and poor HBV knowledge is consistent with findings from a study conducted in Mwanza region, where individuals with higher education demonstrated adequate awareness of viral hepatitis modes of transmission.^11^ Education likely enhances comprehension of health messages and engagement with preventive services, reinforcing the importance of incorporating tailored health literacy interventions within harm-reduction programs.

Shorter duration of methadone treatment also predicted poor knowledge, suggesting that longer engagement in methadone programs increases opportunities for exposure to health education, counselling, and peer learning.^20^ Low economic status further contributed to poor knowledge, aligning with global evidence that economic insecurity constrains access to healthcare information and preventive services.^21^ This finding underscores the need for integrated socioeconomic and health interventions targeting vulnerable PWID populations.

Low risk perception on hepatitis B virus (HBV) infection among people who inject drugs is strongly influenced by low educational attainment and inadequate knowledge about the disease. Individuals with limited formal education often struggle to access and interpret accurate health information, which restricts their understanding of HBV transmission and consequences. Similarly, poor knowledge and low health literacy diminish awareness of personal vulnerability, leading many PWIDs to underestimate their susceptibility to infection. This misperception reduces motivation to engage in preventive measures such as vaccination, regular testing, and the use of sterile injecting equipment.

Recent studies have demonstrated that PWIDs with lower education levels are significantly less likely to recognise HBV as a personal health threat and consequently exhibit weaker preventive behaviours.^11,22^ These findings underscore the critical need for targeted education interventions that enhance HBV-related knowledge and risk perception within harm-reduction programs, thereby promoting more proactive prevention and health-seeking behaviours among PWIDs.

The observed link between poor knowledge and low risk perception reinforces theoretical models such as the Health Belief Model and the Cognitive-Behaviour Framework, which posit that awareness of disease transmission and severity is foundational to perceived susceptibility and behaviour change.^23,24^ Without adequate understanding of HBV transmission routes and prevention, individuals are less likely to internalise their risk or seek protective measures such as vaccination or safe injecting practices. This interconnection calls for integrated educational interventions that combine factual knowledge dissemination with interactive risk communication tailored to literacy levels of PWIDs.

### Implications for Practice and Policy

These findings emphasise the urgent need to strengthen HBV prevention strategies within methadone maintenance and harm-reduction programs in Tanzania. Routine and early HBV counselling should be embedded at program entry, complemented by continuous health education, peer-support models, and visual or participatory approaches suitable for low-literacy populations. Addressing economic and structural barriers through social support and linkage to healthcare services can further improve awareness, vaccination uptake, and long-term health outcomes. Integrating HBV education into existing national harm-reduction and HIV frameworks would provide a cost-effective strategy to reduce the dual burden of infectious diseases among PWIDs.

### Strengths and Limitations

This study provides valuable and timely evidence on hepatitis B virus infection (HBV) related knowledge and risk perception among people who inject drugs (PWIDs), a population that is often underrepresented in research in sub-Saharan Africa. By focusing on individuals enrolled in methadone treatment programs, the study captures insights from a high-risk group that is directly accessible to prevention and care services. The findings offer a strong empirical basis to inform targeted health education, vaccination strategies, and harm-reduction interventions within existing opioid substitution therapy platforms.

However, several limitations must be acknowledged. First, the cross-sectional design restricts causal inference between predictors and outcomes. Second, the use of self-reported data introduces potential social desirability and recall biases, particularly regarding knowledge and perceived risk behaviours. Third, generalizability may be limited to PWIDs enrolled in methadone programs in similar urban settings, as the experiences of community-based or untreated PWIDs might differ. Despite these limitations, the study offers valuable insights to guide targeted educational and preventive interventions for this high-risk population.

## CONCLUSIONS

This study revealed that knowledge and risk perception toward hepatitis B virus (HBV) infection among PWIDs remain alarmingly low. Only one-third of participants demonstrated good knowledge, and merely a quarter perceived themselves at high risk of infection. Low education level, economic disadvantage, and shorter duration of methadone treatment were the strongest predictors of poor HBV knowledge, while low risk perception was significantly associated with both low education and poor knowledge. These findings underscore the urgent need to integrate comprehensive HBV education, counselling, and vaccination awareness into harm-reduction and methadone programs. Interventions should particularly target newly enrolled clients and those with limited formal education or low income to foster accurate risk perception and improve preventive behaviours.
